# Supplementary material for: Evolving scientific discovery by unifying data and background knowledge with AI Hilbert
Source: Nat Commun. 2024 Jul 14;15:5922. doi: 10.1038/s41467-024-50074-w (PMC11247103; doi:10.1038/s41467-024-50074-w)
Supplement: Supplementary file 1 — Supplementary Information [file 41467_2024_50074_MOESM1_ESM.pdf]

Supplementary material

Evolving Scientific Discovery by Unifying Data and Background Knowledge with AI Hilbert

Ryan Cory-Wright[1*] **Cristina Cornelio**[2] **Sanjeeb Dash**[3]

Bachir El Khadir[3] **Lior Horesh**[3]

[1] Department of Analytics, Marketing and Operations,
Imperial College Business School, London, UK
[2] Samsung AI, Cambridge, UK
[3] IBM Thomas J. Watson Research Center, Yorktown Heights, USA
[*] Corresponding Author: r.cory-wright@imperial.ac.uk

1 Additional Related Work

We addressed the most relevant literature in the introduction to the paper. `AI-Hilbert` builds upon two areas of the optimization and discovery literature which are often considered in isolation: sum-of-squares techniques for solving polynomial optimization problems, and data-driven techniques for symbolic discovery. Accordingly, we now review this literature in more detail.

Sum-of-Squares Optimization: Sum-of-squares optimization has been an important component of global optimization methods since the seminal work of Parrilo [34] (see also Lasserre [31]), which combines two key observations. First, sum-of-squares decompositions of multivariate polynomials can be computed via semidefinite optimization, so optimizing over sum-of-squares polynomials is no harder than performing semidefinite optimization. Second, owing to a fundamental result from real algebraic geometry, the Positivstellensatz [29, 39, 35], polynomials of bounded degree defined on basic semialgebraic sets can be certified as non-negative over these sets by representing them as systems of sum-of-squares polynomials. Consequently, optimizing over a real polynomial system is equivalent to solving a (larger) sum-of-squares optimization problem, and thus a tractable convex problem. These observations have allowed an entire field of optimization to blossom; see Blekherman et al. [8], Hall [25] for reviews. However, to our knowledge, no works have proposed using sum-of-squares optimization to discover scientific formulae. The closest works are Clegg et al. [12], who propose using Gröbner bases to design proofs of unsatisfiability, Curmei and Hall [17], who propose a sum-of-squares approach to fitting a polynomial to data under very general constraints on the functional form of the polynomial, e.g., non-negativity of the derivative over a box, Ahmadi and El Khadir [1], who propose learning the behavior of noisy dynamical systems via semialgebraic techniques, and Fawzi et al. [19], who propose learning proofs of optimality of stable set problems by combining reinforcement learning with the Positivstellensatz. However, determining whether polynomial optimization is useful for scientific discovery is, to our knowledge, open.

Data-Driven Approaches to Scientific Discovery: The availability of large amounts of scientific data generated and collected over the past few decades has spurred increasing interest in data-driven methods for scientific discovery that aim to identify symbolic equations that accurately explain high-dimensional datasets. Bongard and Lipson [9] and Schmidt and Lipson [38] proposed using heuristics and genetic programming to discover scientifically meaningful formulae, and implement their approach in the `Eureqa` software system [18]. Other proposed approaches are based on mixed-integer global optimization [3, 14], sparse regression [10, 37, 7], Cylindrical Algebraic Decomposition [22], neural networks [26, 30], and Bayesian Markov Chain Monte Carlo approaches [24]. See [28, 4] for reviews of data-driven scientific discovery in fundamental physics and chemistry.

Data-driven approaches have been shown by several authors to perform well in highly overdetermined settings with limited amounts of noise. For instance, Udrescu et al. [42, 41] proposed a method called `AI-Feynman`, which combines neural networks with physics-based techniques to discover symbolic formulae. Moreover, they constructed a benchmark dataset of 100 scientific laws derived from Richard Feynman's lecture notes [20], with $100,000$ noiseless experimental observations of each scientific law, and demonstrated that while the `Eurequa` system could recover an already impressive 71/100 instances from the data, their approach could recover all one hundred; see Cornelio et al. [13] for a review of scientific discovery systems.

Unfortunately, data-driven approaches to scientific discovery have at least three significant drawbacks. First, they are not data efficient [21] and only reliably recover scientific formulae in overdetermined settings with orders of magnitude more data than a human would likely need to make the same discoveries. Indeed, Matsubara et al. [32] recently argued that the sampling regime used by `AI-Feynman` is unrealistic, because it samples values far from those observable in the real world. Moreover, Cornelio et al. [13] recently benchmarked `AI-Feyman` on 81 of the 100 laws, but with 10 (rather than $100,000$) observations per law, and where each experimental observation is contaminated with a small amount of noise. In this limited data setting, Cornelio et al. [13] found that `AI-Feynman` recovered 40 of the 81 laws considered, whereas [13] were able to recover 49/81 laws using their symbolic regression solver. This performance degradation is a significant issue in practice because scientific data is typically expensive to obtain and scarce and noisy. Second, purely data-driven methods are agnostic to important background information, such as existing literature, that valid scientific formulae should be consistent with unless there is extraordinary experimental evidence that the literature is incorrect. This implies that data-driven methods search over a larger space of laws than is necessary, require more data than a human would need to derive a valid law, and frequently propose laws that are not scientifically meaningful. Third, data-driven methods typically do not provide interpretable explanations for why their discoveries are valid [c.f. 36], which makes diagnosing whether their discoveries are consistent with existing theory challenging.

To account for background theory in scientific discovery, Cornelio et al. [13] recently proposed an approach called `AI-Descartes`, which iteratively generates plausible scientific formulae using a mixed-integer nonlinear symbolic regression solver [see also 3], and tests whether these formulae are derivable from the background knowledge. If they are not, the method provides a set of reasoning-based measures to compute how distant the formulae induced from the data are from the background theory but is unable to recover the correct formulae. This is because their approach induces potential scientific laws from data and subsequently tests the hypothesis against the background theory, rather than learning from axioms and data simultaneously.

2 Comparison with Methods from the Literature

To illustrate our system's capabilities, Table 1 compares `AI-Hilbert` with four approaches from the literature in terms of their ability to recover various scientific laws from background theory and experimental data.

The data for the first two problems comes from real measurements (denoted with "R" in the table)

while for the other problems we rely on simulated data (denoted with "S" in the table). The data is created similarly to the approach used in the literature (see [13] or [42] for more details): we sample ten data points uniformly at random for each problem, independently for each variable in a given continuous range of values. The range is either defined following ranges in real measurements or set artificially (e.g., $(0, 1]$). We then add 1% Gaussian noise to the dependent variable (following [42]), thereby creating small, noisy datasets to resemble real-life data.

We used the background theory provided by Cornelio et al. [13] for the first eight problems and manually extracted the axioms for the remaining ones.

The systems we consider are:

- **AI-Descartes** [13] is a system that combines logical reasoning with symbolic regression to generate meaningful formulae explaining a given phenomenon. It starts by generating a set of candidate models from data alone using sumbolic regression and then validates and ranks them using a background theory. In this way the system ensure that the chosen hypothesis is not only close to the data but also respects the laws already known about the environment, often providing a logical proof of its correctness. However, in the experiments below, we only run the symbolic regression module of AI-Descartes.

- **AI Feynman** [42, 40] is a symbolic regression approach integrating deep learning methodologies with the exploitation of some features commonly found in physics functions including unit presence, utilization of low-order polynomials, and symmetry. The algorithm comprises several modules handling polynomial fitting, exhaustive enumeration, neural network fitting, among other tasks, ultimately generating a list of potential formulas.

- **PySR** [15, 16] employs a combination of regularized evolution, simulated annealing, and gradient-free optimization techniques to search for equations that best match the provided input data.

- **Bayesian Machine Scientist (BMS)** [24] utilizes a Markov chain Monte Carlo approach to traverse the potential model space, leveraging prior expectations learned from a large empirical corpus of mathematical expressions.

The above systems return a score (in some cases this corresponds to the error) and the complexity for each output formula. The systems PySR and Bayesian Machine Scientist return a best candidate. For AI-Descartes and AI-Feynman, we declare success if the correct formula is among the candidates returned, whereas for PySR and Bayesian Machine Scientist, we check if the best candidate formula is correct.

2.1 Experimental results for comparison with prior literature

We now compare `AI-Hilbert` with four methods from the literature in Table 1. Note that all four existing methods that we compare `AI-Hilbert` against require experimental data to successfully discover scientific laws; further, we take results for these methods for the FSRD problems, the Relativistic time dilation problem, and Kepler's third law from [13]. On the other hand, `AI-Hilbert` combines (potentially limited and noisy) data and (potentially corrupted) theory, including the two limiting cases where either only data or only theory are supplied.

`AI-Hilbert` outperforms the existing systems, accurately recovering formulas in all studied problems. Moreover it achieves this with less data, by leveraging relevant background theory. Finally, our method is the only one capable of recovering symbolic constants (while the other systems combine them into single or multiple real values) thereby preserving the meaning and semantics of each individual constant.

We remark that for some methods from the literature, inexact recovery (denoted with a $\checkmark^{\star}$) may occur because these approaches are data-driven, and thus usually cannot derive symbolic constants

associated with scientific laws. Instead, they group information concerning multiple constants into one that is less interpretable. For instance, consider the Compton Scattering problem (3.7), the correct formula is $l_2 = l_1 + \frac{h}{m_e c}(1 - cos(\theta))$ while data-driven methods usually only derive $l_2 = l_1 + k(1 - cos(\theta))$ (where k is a real number). Moreover, sometimes integers are reported with a small numerical error (e.g., 1,001 or 0.999 instead of 1), because Mosek operates with finite precision arithmetic. This could be remedied by using an exact-precision SDP solver [e.g. 33].

Supplementary Table 1: Comparison of AI-Hilbert with state-of-the-art discovery methods in terms of ability to recover scientific laws from background theory and experimental data. First five rows denotes experiments conducted in the main body of the paper.

Data		AI-Hilbert	AI-Descartes [13]	AI-Feynman [42]	PySR [15]	BMS [24]
S	Hagen Poiseuille	✓	✗	✗	✗	✗
S	Gravitational Wave Power	✓	✗	✗	✗	✗
R	Relat. Time Dilat.	✓	✗	✗	✗	✗
R	Kepler's 3 Law	✓	✓*	✗	✓	✗
S	Bell inequalities†	✓	✗	✗	✗	✗
S	[3.1] I.15.10 FSRD	✓	✓*	✗	✗	✗
S	[3.1] I.27.6 FSRD	✓▷	✓*	✓*	✓	✗
S	[3.1] I.34.8 FSRD	✓▷	✓	✓*	✓	✓*
S	[3.1] I.43.16 FSRD	✓	✓*	✓*	✓	✓*
S	[3.1] II.10.9 FSRD	✓	✓*	✓*	✓*	✓*
S	[3.1] II.34.2 FSRD	✓	✓*	✓*	✓*	✓*
S	[3.2] Inelastic Relativ. Collision	✓	✓*	✗	✗	✗
S	[3.3] Decay of Pion◇	✓	✓*	✗	✗	✓*
S	[3.4] Radiation Damping	✓	✗	✗	✓*	✓*
S	[3.5] Escape Velocity	✓	✓	✓*	✓*	✓*
S	[3.6] Hall Effect	✓	✓*	✗	✓*	✓*
S	[3.7] Compton Scattering	✓	✓*	✓*	✓*	✓*

Notation used in table:

✓ denotes the successful recovery of a scientific law.

✗ denotes the failure to recover a scientific law.

✓* denotes recovery up to constants, but not exact recovery.

✓▷ denotes successful recovery when some variables that are potentially not observable are still declared to be observable.

† Inequalities are not supported by the methods from the literature that we benchmarked against.

◇ To generate data for this problem, we varied the mass values of both the pion and the muon. These variations were intended to mimic small differences in the observed values of the masses, rather than altering the actual values across a broad range of values.

3 Discovery of Additional Problems From Background Theory

We now validate `AI-Hilbert` on six problems from Feynman's lectures, considered earlier in AI-Descartes [13]. We also examined six additional problems to demonstrate `AI-Hilbert`'s capacity to rediscover scientific laws from background theory alone. Specifically, in Appendix 3.2, we derive the inelastic relativistic collision law, in Appendix 3.3, we derive the kinetic energy and momentum when a pion at rest decays into a muon and a neutrino, in Appendix 3.4 we derive the light scattering law, in Appendix 3.5 we derive the minimal velocity that enables an object to overcome the gravitational pull of a planet, in Appendix 3.6 we derive the Hall potential of an electrical conductor, and in Appendix 3.7 we derive the Compton scattering formula.

3.1 Problems from FSRD

We consider the background theory provided in in AI-Descartes [13] for the following six problems from Feynman's Lectures: I.15.10, I.27.6, I.34.8, I.43.16, II.10.9 and II.34.2. As shown in the previous table (Table 1), AI-Hilbert obtained the correct symbolic expression for each problem. For the second and third problems, we declared some potentially nonmeasurable variables measurable and permitted AI-Hilbert to search for polynomials incorporating these variables. However, the final polynomial found by AI-Hilbert did not include these nonmeasurable variables.

3.2 Inelastic Relativistic Collision

We consider the problem of computing the momentum and speed of a particle created by colliding a fast-moving particle with a stationary particle. This problem appears within the exercises in the Feynman Lectures on Physics series [20].

We first introduce relevant notation: let m denote the mass of the moving particle, c be the speed of light, $v_m := 4/5c$ denote the speed of the moving particle pre-collision, m_c and v_c denote the mass and speed, respectively, of the composite particle after the collision, p_m and p_c denote the momentum of the moving particle before and after collision, respectively, and E_m, E_r, E_c denote, respectively, the energy of the moving particle before collision, the resting particle before collision and the composite particle after collision.

Then, given that a particle of mass m moving at a speed of $v_m = 4c/5$ collides in elastically with a similar particle at rest, we have the relations

$$p_c = \frac{4m_c v_c c}{\sqrt{3(c^2 - v_c^2)}}, m_c = \frac{4m}{\sqrt{3}}$$

We now derive these relations axiomatically using `AI-Hilbert`, from the following axioms (via appropriate polynomial multipliers):

$$p_m^2(c^2 - v_m^2) - m^2 v_m^2 c^2 = 0 \quad (1)$$

$$E_m^2 - (mc^2)^2 - p_m^2 c^2 = 0 \quad (2)$$

$$E_r - mc^2 = 0 \quad (3)$$

$$E_c^2 - m_c^2 c^4 - p_c^2 c^2 = 0 \quad (4)$$

$$2E_m E_r - E_c^2 + E_m^2 + E_r^2 = 0 \quad (5)$$

$$p_c - p_m = 0 \quad (6)$$

$$v_m^2 - 16/25c^2 = 0 \quad (7)$$

$$p_c^2(c^2 - v_c^2) - m_c^2 v_c^2 c^2 = 0. \quad (8)$$

Equation (1) gives the momentum of the moving particle pre-collision (by rewriting the expression $p_m = \frac{mv}{\sqrt{1-v^2/c^2}}$ as a polynomial equation). Equation (2) models the energy of the moving particle

pre-collision, Equation (3) models the energy of the particle at rest pre-collision, Equation (4) models the energy of the combined particle post-collision, Equation (5) models the conservation of energy (squaring all terms to reduce the degree of the proof certificate; we could instead introduce $E_c - E_m - E_r = 0$ and obtain essentially the same result), Equation (6) models the conservation of momentum, Equation (7) defines the velocity of the moving particle pre-collision (again squaring both sides to reduce the degree of the proof certificate), and Equation (8) defines the momentum of the composite particle.

By requiring that only the terms m, m_c, c appear in our final formula, `AI-Hilbert` provides the following polynomial equality as an output

$$-1.33m_c^4(c^2)^3 + 5.33m^2m_c^2(c^2)^3 + 9.48m^4(c^2)^3 = 0$$

Moreover, this equality can be manipulated to rediscover the two relations at the start of this section. In particular, it follows directly from the equality that $m_c^2 = 16/3m^2$, and hence $m_c = \frac{4m}{\sqrt{3}}$

Moreover, substituting this expression for m_c into Equation (8) and re-arranging yields:

$$p_c = \frac{16mv_cc}{3\sqrt{c^2 - v_c^2}}.$$

3.3 Decay of Pion into Muon and Neutrino

Next, we consider the problem of deriving the kinetic energy and momentum generated when a pion at rest decays into a muon and a neutrino.

We first introduce relevant notation: let m_π, m_μ and m_ν stand for, respectively, the mass of the pion, muon, and neutrino. Further, let p_μ be the momentum of the muon, p_ν be the momentum of the neutrino, and let E_π, E_μ, E_ν be the respective total energy of the pion, the muon, and the neutrino. Given these quantities, the momentum of the neutrino satisfies the relation

$$p_\nu = \frac{m_\pi^2 - m_\mu^2}{2m_\pi}. \tag{9}$$

Accordingly, we now derive this relation by adding together the following background theory axioms with appropriate polynomial multipliers (normalizing in order that $c = 1$ for simplicity):

$$p_\nu - p_\mu = 0 \tag{10}$$
$$E_\pi - m_\pi = 0 \tag{11}$$
$$E_\nu - p_\nu = 0 \tag{12}$$
$$E_\pi - E_\mu - E_\nu = 0 \tag{13}$$
$$E_\mu^2 - p_\mu^2 - m_\mu^2 = 0 \tag{14}$$

where Equation (10) ensures that the decay obeys conservation of momentum, Equation (11) defines the energy of the pion (recall that we set $c = 1$ here), Equation (12) defines the energy of the neutrino (which equals its momentum as it is massless), Equation (13) enforces conservation of energy, and Equation (14) defines the kinematic equation of the muon particle.

We assume that the only measured quantities are p_ν, m_π, m_μ. Then `AI-Hilbert` derives the equality

$$-2m_\pi^2 + 2m_\mu^2 + 4p_\nu m_\pi = 0$$

which is equivalent to the original relation (9).

3.4 Radiation Damping and Light Scattering

We now consider the problem of computing the total amount of energy radiated by a non-relativistic acceleration of a charge.

Denote by S the amount of energy that passes per square meter per second through a surface normal to the radiation, let q_c represent an electric charge accelerating at rate a_p, let r and θ represent, respectively, the distance and angle at which the radiation is observed, and suppose that the charge is oscillating with displacement x_0 at frequency w. Given this notation, we have the natural law:

$$P = \frac{4}{3}\pi q_c^2 x_0^2 w^4 \tag{15}$$

For this problem, we use the following background theory:

$$Sr^2 - q_c^2 a_p^2 sin(\theta)^2 = 0 \tag{16}$$

$$dA - 2\pi r^2 sin(\theta) d\theta = 0 \tag{17}$$

$$P - \int_0^\pi S dA = 0 \tag{18}$$

$$\frac{4}{3} - \int_0^\pi sin(\theta)^3 d\theta = 0 \tag{19}$$

$$a_p^2 - \frac{1}{2} w^4 x_0^2 = 0 \tag{20}$$

where Equation (16) models the rate of radiation of energy, Equation (17) represents a differential of the area of a spherical segment, Equation (18) is the power to rate of radiation relation, Equation (19) is a definite integral from 0 to π, such an integral can be provided as background knowledge, or can be solved by symbolic/numerical integrator, and lastly Equation (20) refers to the average of the acceleration of an oscillating charge over a cycle (squared).

`AI-Hilbert` provides the relation

$$4.0P - 16.76 q_c^2 w^4 x_0^2 = 0$$

which can easily be manipulated to recover Equation (15).

3.5 Escape Velocity

We now consider the problem of deriving the escape velocity of a sphere-shaped planet, that is, the minimal velocity needed for any object attempting to exit a planet to overcome the planet's gravitational pull. Let M denote the mass of the planet, r denote its radius, m denote the mass of the object, v_e denote the escape velocity of the object, K_i and K_f denote the initial and final kinetic energy of the object, U_i and U_f denote the initial and final potential energy of the system, and G denote the universal gravitational constant. We have the relation:

$$v_e = \sqrt{\frac{2GM}{r}} \tag{21}$$

We now recover this scientific law via `AI-Hilbert` from the background theory consisting of the

following polynomial equalities:

$$K_i - \frac{1}{2}mv_e^2 = 0 \tag{22}$$
$$K_f - 0 = 0 \tag{23}$$
$$U_i r + GMm = 0 \tag{24}$$
$$U_f - 0 = 0 \tag{25}$$
$$K_i + U_i - (K_f + U_f) = 0, \tag{26}$$

where Equation (22) models the initial kinetic energy of the object, Equation (23) models the final kinetic energy of the object, Equations (24)–(25) reflect the initial and final potential energy of the body, and Equation (26) models conservation of energy.

Assuming that the only measured quantities are G, M, m, r and v_e. `AI-Hilbert` uncovers the following polynomial relation that is derivable from the background theory:

$$2GMm - mv_e^2 r = 0.$$

This can easily be rearranged to recover Equation (21).

3.6 Hall Effect

The Hall effect produces a potential difference (the Hall voltage, U_H) across an electrical conductor transverse to an electric current in the conductor and to an applied magnetic field perpendicular to the current. Let a metal plate of length L, width h, and depth d be subjected to a potential field across its length dimension. The plate is placed in a homogeneous magnetic field B and perpendicular to the electric field E (oriented with the depth dimension). The electron charges q_e moving at velocity v across the length dimension, will be deflected by the magnetic field (due to a Lorenz force F_m). This will entail an excess of electrons on one side of the plate, and a deficiency on the other side, resulting in an electric field E and respective electric force F_e opposite to the magnetic force. Let the potential difference across the width dimension of the plate be U_H. Finally, let I denote the current, which is defined by the number of electron charges N crossing per time interval dt. The Hall potential is given by the expression:

$$U_H = \frac{hLIB}{Nq_e}. \tag{27}$$

Equation (27) is recoverable from the following background theory via AI-Hilbert:

$$F_m - q_e vB = 0 \tag{28}$$
$$F_e - q_e E = 0 \tag{29}$$
$$F_m - F_e = 0 \tag{30}$$
$$Eh - U_H = 0 \tag{31}$$
$$vdt - L = 0 \tag{32}$$
$$Idt - Nq_e = 0 \tag{33}$$

where Equation (28) models the Lorentz magnetic force, Equation (29) represents the electric force (E is the homogeneous electric field between the upper and lower metal plate), Equation (30) models Newton's third law, Equation (31) relates the electric potential U_H to field E per plate of width h, Equation (32) models the traversal velocity $v = \frac{L}{dt}$ across the plate length L, and Equation (33) models the amount of charge Q, is the current I times dt (can be measured by an amp meter).

We assume the measured variables are L, h, I, B, N, q_e and U_H. `AI-Hilbert` provides the relation

$$q_e^2 U_H L N^2 - q_e B h L^2 I N = 0,$$

which can easily be rearranged to recover Equation (27).

3.7 Compton Scattering

Compton scattering refers to the behavior of high frequency photons as they scatter after collision with a charged particle, usually an electron e in an atom. Specifically, when a photon knocks out a loosely bound electron from the outer valence shells of of an atom (or molecule), a new photon is emitted from the atom traveling at an angle θ to the incoming photon's path. Compton related the shift in photon wavelengths to the scattering angle: $\lambda_2 - \lambda_1 = \frac{h}{m_e c}(1 - \cos\theta)$ where λ_1, λ_2 are the initial and final photon wavelengths, m_e is the electron rest mass, c is the speed of light, and h is Planck's constant.

The following equations give a complete set of axioms needed to derive Compton's formula. Let E_r be the energy of the electron at rest (i.e., an electron bound to an atom), let E_m be the energy of the moving electron (after it is knocked out from the atom). Let E_1, E_2 be the initial and final photon energies, f_1, f_2 be the initial and final photon frequencies, and p_1, p_2 be the initial and final photon momentum values. The first constraint is just the expression for conservation of energy. The second and third constraints give the photon energy in terms of frequency. The subsequent two constraints give photon momentum in terms of frequency, and the two constraints after that relate wavelength and frequency. The next constraint is the mass-energy equivalence of the electron at rest, while the constraint after that gives the energy of the moving electron via the relativistic energy–momentum relation. The last equation is a restatement of conservation of momentum (after squaring).

$$E_1 + E_r - E_2 - E_m = 0 \tag{34}$$
$$E_1 - hf_1 = 0 \tag{35}$$
$$E_2 - hf_2 = 0 \tag{36}$$
$$p_1 c - hf_1 = 0 \tag{37}$$
$$p_2 c - hf_2 = 0 \tag{38}$$
$$\lambda_1 f_1 - c = 0 \tag{39}$$
$$\lambda_2 f_2 - c = 0 \tag{40}$$
$$E_r - m_e c^2 = 0 \tag{41}$$
$$E_m^2 - pe_2^2 c^2 - m_e^2 c^4 = 0 \tag{42}$$
$$pe_2^2 - p_1^2 - p_2^2 + 2p_1 p_2 \cos\theta = 0 \tag{43}$$

If we assume that the only measured variables are $h, c, m_e, \lambda_1, \lambda_2$ and $\cos\theta$, AI Hilbert provides the relationship

$$h^2c^2 - h^2c^2\cos^2\theta - \lambda_2 h m_e c^3 + \lambda_1 h m_e c^3 - \lambda_2 h m_e c^3 \cos\theta + \lambda_1 h m_e c^3 \cos\theta = 0,$$

which becomes, after rearranging terms

$$(\lambda_2 - \lambda_1) h m_e c^3 (1 + \cos\theta) = h^2 c^2 (1 - \cos^2\theta).$$

Dividing both sides by $h m_e c^3(1 + \cos\theta)$, we get the desired relationship. An important assumption we made above is that measurements of $\cos\theta$ are available and not just of θ. This is reasonable in many areas of physics as we often take the component of velocity, force etc. in a direction at an angle θ to the direction of the velocity, force etc. It is also reasonable to assume that h, c, m_e are measured quantities, as these are known quantities.

4 Supplementary Material From Methods

4.1 Impact of Background Theory on Amount of Data Needed to Discover Scientific Laws

In the introduction, we suggested that providing a partially complete background theory expressible as polynomial equalities and inequalities may accelerate the scientific discovery process by decreasing the amount of data required to recover a scientific law with high probability. We now justify our claim in the introduction, by reviewing examples from the machine learning literature, which may be viewed as special cases of scientific discovery, where including relevant background theory decreases the number of data points required to recover a scientific law with high probability. The first two examples involve discovery settings where the ground truth is known to be sparse, and imposing a sparsity constraint on the discovered law reduces the amount of data required to recover the law with high probability. Note that these examples are due to [23, 2, 11]. However, the similarities between them strongly suggest that similar relations between the amount of background theory introduced and the amount of data required for scientific discovery also hold in other contexts.

Example 1 *Sparse Linear Regression [23]*
Consider a sparse high-dimensional regression model where we aim to recover a τ-sparse regression model $\boldsymbol{\beta}^\star \in \mathbb{R}^p$ given access to n noisy linear observations of the form $\boldsymbol{Y} = \boldsymbol{X}\boldsymbol{\beta}^\star + \boldsymbol{W} \in \mathbb{R}^n$, where $X_{i,j} \overset{\text{iid}}{\sim} \mathcal{N}(0,1)$ and $W_i \overset{\text{iid}}{\sim} \mathcal{N}(0,\sigma^2)$ for some parameter $\sigma > 0$, it is known that $\boldsymbol{\beta}^\star$ is a τ-sparse vector with binary coefficients, and $n \ll p$ with $p \to \infty$. Then, it is information-theoretically impossible to recover $\boldsymbol{\beta}$ if $n \leq \Theta\left(\frac{2\tau \log p}{\log\left(1+\frac{2\tau}{\sigma^2}\right)}\right)$ [43]. On the other hand, for $n \geq \Theta\left(\frac{2\tau \log p}{\log\left(1+\frac{2\tau}{\sigma^2}\right)}\right)$, the (unique) optimal solution of the polynomial optimization problem (expressible as a binary problem)

$$\min_{\boldsymbol{\beta} \in \mathbb{R}^p} \quad \|\boldsymbol{Y} - \boldsymbol{X}\boldsymbol{\beta}\|_2^2 \text{ s.t. } \beta_i^2 = \beta_i \ \forall i \in [p], \sum_{i \in [p]} \beta_i = \tau \tag{44}$$

is such that

$$\frac{1}{\tau}\|\boldsymbol{\beta}^\star - \boldsymbol{\beta}\|_0 \to 0 \tag{45}$$

with high probability as $\tau \to \infty$. On the other hand, for any $\lambda > 0$, the optimal solution of the popular Lasso method

$$\min_{\boldsymbol{\beta} \in \mathbb{R}^p} \quad \|\boldsymbol{Y} - \boldsymbol{X}\boldsymbol{\beta}\|_2^2 + \lambda\|\boldsymbol{\beta}\|_1 \tag{46}$$

only recovers $\boldsymbol{\beta}^\star$ with high probability when $n \geq \Theta(2\tau + \sigma^2)\log p$. We remind the reader that

$$\Theta(2\tau + \sigma^2)\log p > \Theta\left(\frac{2\tau \log p}{\log\left(1 + \frac{2\tau}{\sigma^2}\right)}\right). \tag{47}$$

Example 2 *Sparse Principal Component Analysis [2]*
Consider a sparse principal component analysis setting where we aim to recover a τ-sparse binary vector $\boldsymbol{x}^\star \in \mathbb{R}^n$ given an observed matrix $\boldsymbol{Y} = \frac{\lambda}{\tau}\boldsymbol{x}^\star \boldsymbol{x}^{\star\top} + \boldsymbol{W}$, where $\boldsymbol{W}$ is a GOE(n) matrix, i.e., is a symmetric matrix with on-diagonal entries taking values $W_{i,i} \overset{\text{iid}}{\sim} \mathcal{N}(0, \frac{2}{n})$ and off-diagonal entries taking values $W_{i,j} \overset{\text{iid}}{\sim} \mathcal{N}(0, \frac{1}{n})$, and $\lambda > 0$ is the signal-to-noise ratio. Let λ, τ possibly depend on n, and set $1 \ll \tau \ll n$ as $n \to \infty$. Then:

- *Recovery of $\boldsymbol{x}^\star$ is information-theoretically impossible when $\lambda \ll \frac{\sqrt{\tau}}{\sqrt{n}}$, with high probability.*

- *The (mixed-integer representable) polynomial optimization problem*

$$\max_{\boldsymbol{x}\in\mathbb{R}^n} \quad \boldsymbol{x}^\top \boldsymbol{Y}\boldsymbol{x} \text{ s.t. } x_i^2 = x_i \ \forall i \in [n], \ \sum_{i\in[n]} x_i = \tau \tag{48}$$

  achieves exact recovery with high probability when $\lambda \gg \frac{\sqrt{\tau}}{\sqrt{n}}$.

- *The diagonal thresholding algorithm of [27] recovers* $\boldsymbol{x}^\star$ *with high probability if* $\lambda \gg \frac{\tau}{\sqrt{n}}$.

- *The vanilla PCA method, which disregards backgrounds theory encoded via a sparsity constraint and solves the polynomial optimization problem*

$$\max_{\boldsymbol{x}\in\mathbb{R}^n} \quad \boldsymbol{x}^\top \boldsymbol{Y}\boldsymbol{x} \text{ s.t. } \|\boldsymbol{x}\|_2^2 = \tau \tag{49}$$

  fails to recover $\boldsymbol{x}^\star$ *with high probability when* $\lambda > 1$.

Example 3 *Low-Rank Matrix Completion [11]*
Consider a low-rank matrix completion setting where we aim to recover a fixed rank r $n \times n$ *matrix* $\boldsymbol{A}$ *given a uniform random sample of its entries* $\Omega \subseteq [n] \times [n]$ *of size* m, *where* $\boldsymbol{A}$ *satisfies the mutual incoherence property of [11] with constant* μ. *Then:*

- *Recovery of* $\boldsymbol{A}$ *is information-theoretically impossible when* $m \leq \Theta(nr \log n)$, *because there are infinitely many rank-r matrices that match all observed entries perfectly [11].*

- *The polynomial optimization problem [c.f. 6, 5]*

$$\min_{\boldsymbol{X}\in\mathbb{R}^{n\times n}, \boldsymbol{Y}\in\mathcal{S}^n} \quad \sum_{(i,j)\in\Omega} (X_{i,j} - A_{i,j})^2 \text{ s.t. } \boldsymbol{Y}\boldsymbol{X} = \boldsymbol{X}, \boldsymbol{Y}^2 = \boldsymbol{Y}, \operatorname{tr}(\boldsymbol{Y}) = r \tag{50}$$

  achieves exact recovery with high probability provided $m \geq \Theta(nr \log n)$.

- *The nuclear norm relaxation of [11] recovers* $\boldsymbol{A}$ *with high probability provided* $m \geq \Theta(n^{6/5} r \log n)$.

- *The naive approach of disregarding the rank constraint and solving*

$$\min_{\boldsymbol{X}\in\mathbb{R}^{n\times n}} \sum_{(i,j)\in\Omega} (X_{i,j} - A_{i,j})^2, \tag{51}$$

  which admits the solution $X_{i,j} = 0$ *if* $(i,j) \notin \Omega$, *fails to recover* $\boldsymbol{A}$ *with high probability provided* $m < n^2$.

The above examples are admittedly more stylized than many scientific discovery settings that arise in practice. Nonetheless, they reveal that in certain circumstances, encoding relevant background provably reduces the amount of data required to recover a scientific law with high probability. This agrees with intuition: if we provide a complete background theory that can be manipulated to recover the scientific law, then, as discussed in the next section, we require no data to recover a scientific law. On the other hand, if we provide no background theory we may require a significant amount of data to recover a law. Therefore, providing relevant background theory that constrains the space of derivable scientific laws should decrease the amount of data needed to recover a scientific law with high confidence. This observation highlights the value of embedding relevant background theory within the scientific discovery process.

4.2 Implementation Details

We now illustrate the lower-level implementation of `AI-Hilbert` via a synthetic example where the axioms are assumed to be consistent and complete. Consider a semialgebraic system in two real variables x and y which comprises the axioms:

$$x^2 + y^2 - 2 = 0, \tag{52}$$

$$y - x^3 = 0, \tag{53}$$

where we let $h_1(x, y) = 0$ and $h_2(x, y) = 0$ denote Equations (52)–(53), respectively. These axioms can be viewed as being true in a subdomain of $\mathbb{R}^2$, i.e., when $|x| = 1$.

Let the set $S = \{(1, 1), (-1, -1)\}$ contain all points satisfying equations (52)–(53). If

$$q(x, y) = \beta_1(x, y)h_1(x, y) + \beta_2(x, y)h_2(x, y), \tag{54}$$

where β_1, β_2 are polynomials in x, y, then q is a polynomial that vanishes on S. On the other hand, to certify that a polynomial $q(x, y)$ vanishes on S, we search for a polynomial $q(x, y)$ such that $q(x, y) = g(x, y)p(x, y)$ and q satisfies the expression in (54). For example, $q(x, y) = x - y$ vanishes on S; setting $\beta_1 = -\frac{1}{2}x, \beta_2 = -1$, we have

$$q = \beta_1 h_1 + \beta_2 h_2 = x - y + \frac{1}{2}(x^3 - xy^2) = (x - y)(1 + \frac{1}{2}x(x + y)). \tag{55}$$

Suppose we wish to find a polynomial function $q(x, y)$ that vanishes on S and explains the dataset

$$\bar{\boldsymbol{x}} = \begin{bmatrix} .5 & .5 \\ -2 & -2 \\ 3 & 3 \\ -3 & -3 \end{bmatrix}$$

where the columns are observed values of x and y, respectively, and each row represents a datapoint. Note that these data observations are noiseless observations from the polynomial $f(x, y) = x - y$, which we aim to recover. Then we search for q satisfying (54) that fits $\bar{\boldsymbol{x}}$.

We assume that β_1, β_2, q are unknown polynomials that are comprised of monomials of degree at most 3, i.e., the ten monomials in the vector

$$\text{mon} = (1, y, y^2, y^3, x, xy, xy^2, x^2, x^2y, x^3), \tag{56}$$

where $\text{mon}_1 = 1, \text{mon}_2 = y, \ldots, \text{mon}_{10} = x^3$. Let

$$\beta_1(x, y) = \sum_{j=1}^{10} a_j \text{mon}_j,\ \beta_2(x, y) = \sum_{j=1}^{10} b_j \text{mon}_j,\ \text{and } q(x, y) = \sum_{j=1}^{10} c_j \text{mon}_j. \tag{57}$$

Further, let $\boldsymbol{v}$ denote the vector $(a_1, \ldots, a_{10}, b_1, \ldots, b_{10}, c_1, \ldots, c_{10})$, $\bar{\boldsymbol{x}}_i$ denote the ith row of $\bar{\boldsymbol{x}}$, and $q(\bar{\boldsymbol{x}}_i)$ be the value of the polynomial q evaluated at the point $(\bar{x}, \bar{y}) = \bar{\boldsymbol{x}}_i$. Note that $q(\bar{\boldsymbol{x}}_i) = \sum_{j=1}^{10} c_j \text{mon}_j(\bar{\boldsymbol{x}}_i)$ is a linear function of the unknowns $c_1, \ldots, c_{10}$.

If $\bar{\boldsymbol{x}}_i$ is a noiseless experimental observation, we should have $q(\bar{\boldsymbol{x}}_i) = 0$. Accordingly, we interpret any nonzero value of $|q(\bar{\boldsymbol{x}}_i)|$ as the error when q is evaluated at $\bar{\boldsymbol{x}}_i$, and aim to minimize this error when selecting q. Equation (54) implies the following linear equations in the variables a_i, b_i, c_i, which are obtained by equating the coefficients of the monomials in Equation (54):

$$c_1 = -2a_1 \quad \text{[coef. of 1]} \tag{58}$$

$$c_2 = -2a_2 + b_1 \quad \text{[coef. of } y] \tag{59}$$

$$c_3 = a_1 - 2a_3 + b_2 \quad \text{[coef. of } y^2] \tag{60}$$

$$\vdots$$

$$0 = a_3 + b_4 \quad \text{[coef. of } xy^3] \tag{61}$$

Let these constraints be denoted by $Av = \mathbf{0}$, and let m, the number of data points, be an integer between 1 and 4. Then, we solve the linear optimization problem

$$\begin{aligned} \min \quad & 100 \left(\sum_{i=1}^{m} t_i\right) + \sum_{j=1}^{10} w_j & & (62) \\ \text{s.t} \quad & t_i \geq q(\bar{\boldsymbol{x}}_{\boldsymbol{i}}) & i = 1, \ldots, m \\ & t_i \geq -q(\bar{\boldsymbol{x}}_{\boldsymbol{i}}) & i = 1, \ldots, m \\ & w_j \geq c_j & j = 1, \ldots, 10 \\ & w_j \geq -c_j & j = 1, \ldots, 10 \\ & Av = \mathbf{0} \\ & \sum_{j=4}^{10} c_j = 1 \\ & v \in \mathbb{R}^{30}, t_i, w_j \geq 0 & i = 1, \ldots, d, j = 1, \ldots, 10. \end{aligned}$$

In Problem (62), the first two constraints imply that $t_i \geq |q(\bar{\boldsymbol{x}}_{\boldsymbol{i}})|$; the third and fourth force $w_i \geq |c_i|$. The second-to-last constraint forces a monomial containing x to be present in q to avoid the trivial solution $q \equiv 0$. We sometimes use a different right-hand-side value to get non-fractional c_i values. Thus, the optimization problem above searches for a polynomial q that minimizes a weighted combination of the L_1-error of q and the L_1-coefficient norm of q. The latter term is a regularization term incentivizing a sparse q.

If we set $m = 1$ and solve (62), we obtain the (correct) function $q(x, y) = x - y$ in (62). In other words, a single data point from $\bar{\boldsymbol{x}}$ suffices to "recover" $x - y$ with the choice of objective in (62).

However, let LPF be the linear optimization problem obtained by dropping the second term in the objective in (62) (and not incentivizing sparsity). If we solve LPF with $m = 1$, we get $q' = 4x^3 - x^2 - y^2 - 4y + 2$ as a solution. This is not a multiple of $(x - y)$ but vanishes at the points in S and the first datapoint in $\mathcal{D}$. We need $m = 2$ (and use two datapoints) before we get

$$q' = y^3 + x^2 y - 2xy^2 + 4x - 4y = (x - y)\big((x - y)^2 + 4\big). \tag{63}$$

This illustrates the role of sparsity and regularization in reducing the amount of data required to recover a scientific law. Note that $(x - y)^2 + 4$ is strictly positive for all real x, y, and therefore $q' = 0$ on S indicates that $x - y = 0$ on S.

Further, if we drop the constraints $Av = \mathbf{0}$ and allow q to be an arbitrary degree-3 polynomial in x, y (and not equal to $\beta_1 h_1 + \beta_2 h_2$), then we cannot recover a multiple of $x - y$ until we set $m = 4$ and use all datapoints in $\bar{\boldsymbol{x}}$. In the latter case, we get $q = x^3 - y^3$. This highlights the value of background theory in restricting the space of feasible scientific laws and reducing the amount of data required to recover a scientific law.

If the only measured variable is x, then we would try to eliminate y in the final formula. Multiplying (53) by $y + x^3$ and subtracting the result from (52), we get the expression $x^2 + x^6 - 2 = 0$. This satisfies the x-components of points in S. Sometimes, there may be a unique way of eliminating variables and getting a formula on the measured variables.

Supplementary References

[1] A. A. Ahmadi and B. E. Khadir. Learning dynamical systems with side information. *SIAM Review*, 65(1):183–223, 2023.

[2] G. B. Arous, A. S. Wein, and I. Zadik. Free energy wells and overlap gap property in sparse PCA. In *Conference on Learning Theory*, pages 479–482. PMLR, 2020.

[3] V. Austel, S. Dash, O. Gunluk, L. Horesh, L. Liberti, G. Nannicini, and B. Schieber. Globally optimal symbolic regression. *arXiv preprint arXiv:1710.10720*, 2017.

[4] Z. J. Baum, X. Yu, P. Y. Ayala, Y. Zhao, S. P. Watkins, and Q. Zhou. Artificial intelligence in chemistry: current trends and future directions. *Journal of Chemical Information and Modeling*, 61(7):3197–3212, 2021.

[5] D. Bertsimas, R. Cory-Wright, S. Lo, and J. Pauphilet. Optimal low-rank matrix completion: Semidefinite relaxations and eigenvector disjunctions. *arXiv preprint arXiv:2305.12292*, 2023.

[6] D. Bertsimas, R. Cory-Wright, and J. Pauphilet. Mixed-projection conic optimization: A new paradigm for modeling rank constraints. *Operations Research*, 70(6):3321–3344, 2022.

[7] D. Bertsimas and W. Gurnee. Learning sparse nonlinear dynamics via mixed-integer optimization. *Nonlinear Dynamics*, pages 1–20, 2023.

[8] G. Blekherman, P. A. Parrilo, and R. R. Thomas. *Semidefinite optimization and convex algebraic geometry*. SIAM, 2012.

[9] J. Bongard and H. Lipson. Automated reverse engineering of nonlinear dynamical systems. *Proceedings of the National Academy of Sciences*, 104(24):9943–9948, 2007.

[10] S. L. Brunton, J. L. Proctor, and J. N. Kutz. Discovering governing equations from data by sparse identification of nonlinear dynamical systems. *Proceedings of the National Academy of Sciences*, 113(15):3932–3937, 2016.

[11] E. J. Candes and Y. Plan. Matrix completion with noise. *Proceedings of the IEEE*, 98(6):925–936, 2010.

[12] M. Clegg, J. Edmonds, and R. Impagliazzo. Using the Gröebner basis algorithm to find proofs of unsatisfiability. In *Proceedings of the twenty-eighth annual ACM symposium on Theory of computing*, pages 174–183, 1996.

[13] C. Cornelio, S. Dash, V. Austel, T. Josephson, J. Goncalves, K. Clarkson, N. Megiddo, B. E. Khadir, and L. Horesh. Combining data and theory for derivable scientific discovery with AI-Descartes. *Nature Communications*, 14(1777), 2023.

[14] A. Cozad and N. V. Sahinidis. A global MINLP approach to symbolic regression. *Mathematical Programming*, 170:97–119, 2018.

[15] M. Cranmer. PySR: Fast & parallelized symbolic regression in Python/Julia. `doi.org/10.5281/zenodo.4041459`, Sept. 2020.

[16] M. Cranmer, A. Sanchez-Gonzalez, P. Battaglia, R. Xu, K. Cranmer, D. Spergel, and S. Ho. Discovering symbolic models from deep learning with inductive biases. *NeurIPS 2020*, 2020.

[17] M. Curmei and G. Hall. Shape-constrained regression using sum of squares polynomials. *Operations Research*, 2023.

[18] R. Dubčáková. Eureqa: software review. *Genetic Programming and Evolvable Machines*, 12:173–178, 2011.

[19] A. Fawzi, M. Malinowski, H. Fawzi, and O. Fawzi. Learning dynamic polynomial proofs. *Advances in Neural Information Processing Systems*, 32, 2019.

[20] R. P. Feynman, R. B. Leighton, and M. Sands. The Feynman lectures on physics; vol. i. *American Journal of Physics*, 33(9):750–752, 1965.

[21] N. Fujinuma, B. DeCost, J. Hattrick-Simpers, and S. E. Lofland. Why big data and compute are not necessarily the path to big materials science. *Communications Materials*, 3(1):59, 2022.

[22] N. Fulton, S. Mitsch, J.-D. Quesel, M. Völp, and A. Platzer. KeYmaera X: An axiomatic tactical theorem prover for hybrid systems. In *Automated Deduction-CADE-25: 25th International Conference on Automated Deduction, Berlin, Germany, August 1-7, 2015, Proceedings 25*, pages 527–538. Springer, 2015.

[23] D. Gamarnik and I. Zadik. High dimensional regression with binary coefficients. estimating squared error and a phase transtition. In *Conference on Learning Theory*, pages 948–953. PMLR, 2017.

[24] R. Guimerà, I. Reichardt, A. Aguilar-Mogas, F. A. Massucci, M. Miranda, J. Pallarès, and M. Sales-Pardo. A Bayesian machine scientist to aid in the solution of challenging scientific problems. *Science Advances*, 6(5):eaav6971, 2020.

[25] G. Hall. Applications of sums of squares polynomials. In P. Parrilo and R. Thomas, editors, *Sum of Squares: Theory and Applications*, volume 77. Proceedings of Symposia in Applied Mathematics, 2020.

[26] R. Iten, T. Metger, H. Wilming, L. Del Rio, and R. Renner. Discovering physical concepts with neural networks. *Physical Review Letters*, 124(1):010508, 2020.

[27] I. M. Johnstone and A. Y. Lu. On consistency and sparsity for principal components analysis in high dimensions. *Journal of the American Statistical Association*, 104(486):682–693, 2009.

[28] G. Karagiorgi, G. Kasieczka, S. Kravitz, B. Nachman, and D. Shih. Machine learning in the search for new fundamental physics. *Nature Reviews Physics*, 4(6):399–412, 2022.

[29] J.-L. Krivine. Anneaux préordonnés. *Journal d'analyse mathématique*, 12:p–307, 1964.

[30] M. Landajuela, C. S. Lee, J. Yang, R. Glatt, C. P. Santiago, I. Aravena, T. Mundhenk, G. Mulcahy, and B. K. Petersen. A unified framework for deep symbolic regression. *Advances in Neural Information Processing Systems*, 35:33985–33998, 2022.

[31] J. B. Lasserre. Global optimization with polynomials and the problem of moments. *SIAM Journal on Optimization*, 11(3):796–817, 2001.

[32] Y. Matsubara, N. Chiba, R. Igarashi, and Y. Ushiku. SRSD: Rethinking datasets of symbolic regression for scientific discovery. In *NeurIPS 2022 AI for Science: Progress and Promises*, 2022.

[33] S. Naldi. Solving rank-constrained semidefinite programs in exact arithmetic. In *Proceedings of the ACM on International Symposium on Symbolic and Algebraic Computation*, pages 357–364, 2016.

[34] P. A. Parrilo. Semidefinite programming relaxations for semialgebraic problems. *Mathematical Programming*, 96(2):293–320, 2003.

[35] M. Putinar. Positive polynomials on compact semi-algebraic sets. *Indiana University Mathematics Journal*, 42(3):969–984, 1993.

[36] C. Rudin. Stop explaining black box machine learning models for high stakes decisions and use interpretable models instead. *Nature Machine Intelligence*, 1(5):206–215, 2019.

[37] S. H. Rudy, S. L. Brunton, J. L. Proctor, and J. N. Kutz. Data-driven discovery of partial differential equations. *Science Advances*, 3(4):e1602614, 2017.

[38] M. Schmidt and H. Lipson. Distilling free-form natural laws from experimental data. *Science*, 324(5923):81–85, 2009.

[39] G. Stengle. A Nullstellensatz and a Positivstellensatz in semialgebraic geometry. *Mathematische Annalen*, 207(2):87–97, 1974.

[40] S. Udrescu, A. Tan, J. Feng, O. Neto, T. Wu, and M. Tegmark. AI Feynman 2.0: Pareto-optimal symbolic regression exploiting graph modularity. In H. Larochelle, M. Ranzato, R. Hadsell, M. Balcan, and H. Lin, editors, *Advances in Neural Information Processing Systems 33: Annual Conference on Neural Information Processing Systems 2020, NeurIPS 2020, December 6-12, 2020, virtual*, 2020.

[41] S.-M. Udrescu, A. Tan, J. Feng, O. Neto, T. Wu, and M. Tegmark. AI Feynman 2.0: Pareto-optimal symbolic regression exploiting graph modularity. *Advances in Neural Information Processing Systems*, 33:4860–4871, 2020.

[42] S.-M. Udrescu and M. Tegmark. AI Feynman: A physics-inspired method for symbolic regression. *Science Advances*, 6(16), 2020.

[43] W. Wang, M. J. Wainwright, and K. Ramchandran. Information-theoretic limits on sparse signal recovery: Dense versus sparse measurement matrices. *IEEE Transactions on Information Theory*, 56(6):2967–2979, 2010.
